# Is Raman Spectroscopy of Fingernails a Promising Tool for Diagnosing Systemic and Dermatological Diseases in Adult and Pediatric Populations?

**DOI:** 10.3390/medicina60081283

**Published:** 2024-08-09

**Authors:** Teresa Tabasz, Natalia Szymańska, Katarzyna Bąk-Drabik, Aleksandra Damasiewicz-Bodzek, Agnieszka Nowak

**Affiliations:** 1 Faculty of Medical Sciences in Zabrze, Students Association, Medical University of Silesia, 41-808 Katowice, Poland; ttabasz26@gmail.com (T.T.); natalia.szymanska97@gmail.com (N.S.); 2 Department of Paediatrics, Faculty of Medical Sciences in Zabrze, Medical University of Silesia, 41-808 Katowice, Poland; 3 Department of Chemistry, Faculty of Medical Sciences in Zabrze, Medical University of Silesia, Jordana 19, 41-808 Katowice, Poland; aleksandra.bodzek@sum.edu.pl (A.D.-B.); agnieszkanowak@sum.edu.pl (A.N.)

**Keywords:** Raman spectroscopy, fingernails, adults, children

## Abstract

*Background*: Raman spectroscopy is a well-known tool used in criminology, molecular biology, and histology. It is also applied to diagnose bone mineral disorders by taking advantage of the similarity of the structure of keratin and bone collagen. Raman spectroscopy can also be used in dermatology and diabetology. The purpose of the present review is to critically evaluate the available research about the use of Raman spectroscopy in the mentioned areas of medicine. *Methodology*: PubMed was searched for peer-reviewed articles on the subject of use of Raman spectroscopy in bone mineral disorders, dermatology, and diabetes mellitus. *Results*: Nail keratin and bone collagen are related structural proteins that require disulfide bond for structural stability. Therefore, Raman spectroscopy of keratin may have potential as a diagnostic tool for screening bone quality and distinguishing patients at risk of fracture for reasons different from low bone mineral density (BMD) in the adult women population. Raman spectroscopy can also investigate the changes in keratin’s structure in nails affected by onychomycosis and distinguish between healthy and onychomycosis nail samples. It could also reduce the need for nail biopsy by distinguishing between dermatophytic and non-dermatophytic agents of onychomycosis. Additionally, Raman spectroscopy could expedite the diagnostic process in psoriasis (by assessing the secondary structure of keratin) and in diabetes mellitus (by examining the protein glycation level). *Conclusions*: In adult populations, Raman spectroscopy is a promising and safe method for assessing the structure of fingernails. However, data are scarce in the pediatric population; therefore, more studies are required in children.

## 1. Introduction

Examination of the fingernails may reveal many abnormalities. Some are well-known symptoms of underlying systemic diseases. Examples of the morphological changes visible to the naked eye are onychomycosis (fungal infection of the nails), finger clubbing (a sign of chronic obstructive pulmonary disease or cardiac disease), Beau lines (in Raynaud disease and pemphigus), and pincer nails (in psoriasis, Kawasaki disease) [1]. As those abnormalities can be detected macroscopically, it is expected that the structure or chemical compositions of the nails also changes [2]. The visible changes in nails and the resulting structural disturbances suggest that methods, such as Raman spectroscopy, could reveal more information about abnormalities associated with systemic diseases. The diagnostics of nails are important in children, where we should avoid methods based on ionizing radiation or methods causing pain and stress. The prevalence of nail disorders varies among different populations. A study published in Turkey [3] has determined the incidence rate of nail disorders to be 4.4% in pediatric patients. These patients are more susceptible to bacterial and viral diseases and are less likely to experience fungal infection of the nail apparatus. Acquired nail conditions observed in children are similar to those of adults. Acquired nail disorders manifest more often in pediatric patients with underlying systemic disorders [4].

Raman spectroscopy is a well-known and widely-used tool. It is used in gemology [5], and in criminology to confirm the presence of drugs and explosives [6]. Raman spectroscopy can also sense biomolecules, like DNA, proteins, lipids, and carbohydrates. There is some evidence that this method can detect cancer in various tissues [7,8,9,10,11,12,13,14]. Raman spectroscopy can be used to distinguish children with inflammatory bowel disease (IBD) from non-IBD ones and, among IBD patients, to differentiate between ulcerative colitis (UC) and Crohn’s disease (CD) by detecting the changes in secondary structure in the protein extract from fecal samples [15]. Raman spectral characterization of urine could also differentiate between acute kidney injury (AKI) patients and non-AKI patients for future kidney disease control [16]. This approach was also used to diagnose active and latent tuberculosis infection [17]. Raman spectroscopy is an accessible analysis method of protein, lipid, and water in samples of intact skin, hair, and nail. It is a non-destructive analytical method for determining the structure and conformation of chemical compounds. No sample preparation or pre-treatment is required [18]. This review discusses whether Raman spectroscopy could be a promising non-invasive tool to assess adult and pediatric patient health by examining their fingernails.

## 2. Structure of Healthy Nails in Adults and Children

The nail consists of a hard, flat nail plate, which is the final product of the nail matrix [19]. The nail plate is in constant contact with the periosteum of the phalangeal bone; therefore, both physiological and pathological processes occurring in blood and bones may affect the mineral composition of the nail plate, and, consequently, the nail plate may be a good predictor of metabolic changes taking place in the body [20,21]. The average nail plate thickness is 0.5 mm in women and 0.6 mm in men [22]. The main chemical component of the human nail is keratin, a scleroprotein containing a significant amount of sulfur [19]. The nail is produced by a specialized area of the epidermis, the nail matrix, from differentiated anucleate keratinocytes [23]. The main protein component of the human nail is keratin. Its cysteine content determines disulfide (S-S) bonds which are crucial for the structural integrity of nails. Keratin and collagen undergo non-enzymatic and post-translational modifications that can be detected with the aid of Raman spectroscopy. Using Raman spectroscopy, we can take a measurement of the protein sulfation degree in fingernails. Detection of sulfur-containing amino acids (cysteine, methionine), as well as S-S bonds, is possible [24]. Additionally, the nail contains other elements, such as calcium (Ca), magnesium (Mg), sodium (Na), potassium (K), iron (Fe), copper (Cu), zinc (Zn), and trace amounts of chromium (Cr), selenium (Se), gold (Au), mercury (Hg), silver (Ag), and cobalt (Co) [25]. The Ca, Mg, and Fe concentrations in fingernails are significantly lower in men than in women. Furthermore, positive correlations were found between husband and wife, son and father or mother, and mother and daughter for several fingernail elements. The similar eating habits and lifestyles of such couples may explain these correlations [26]. Studies have also evaluated the concentrations of minerals, such as zinc (Zn) and selenium (Se), in toenails. No association was discovered between the toenail Zn levels and the risk of acute myocardial infarction [27], contrary to low selenium and cerium levels [28,29]. Patients with osteogenesis imperfecta showed higher levels of Zn and a significantly lower ratio of Ca/Zn and Mg/Zn in fingernails compared to normal controls [30]. Nails provide valid estimations for ranking subjects according to long-term trace element intake. Consequently, nail clippings have been utilized in studies to detect exposure to heavy metals [31], detect dietary deficiencies, such as iron deficiency [32], and to examine the relationship between the concentration of fingernail microelements with coronary heart disease and hypertension [33].

Findings concerning the pediatric population are scarce. Levels of copper, zinc, magnesium, selenium, lead, and mercury were assessed in the nails of children with autism [34]. Nails of adolescents with different degrees of airway inflammation were analyzed to evaluate the association between a deficiency of trace elements and an increased amount of exhaled nitric oxide (FeNO) [35,36]. In obese patients, a possible association between fingernail selenium levels and risk of obesity was assessed [37].

Nail clippings are easy to collect in a non-invasive way and can be easily stored at room temperature [38]. The nail plate grows continuously throughout an individual’s lifetime, beginning in the nail matrix and growing out constantly over the nail bed. Other cutaneous appendages, such as hair, grow in cycles [39]. Fingernail plates develop at a rate of 3.5 mm per month. Substances, such as medication, pollutants, and biomarkers, may be deposited in nail tissue [20]. The nails of the thumb and the little finger grow slower and more gradually than other fingernails. Ergo, they can have a different mineral content than other fingernails. Therefore, in clinical studies, it is necessary to record the finger utilized for gathering the nail plate samples [24]. The nail growth rate in children is similar to that observed in young adults, with the fastest values of nail growth (1.5 mm per day) occurring between the ages of 10 and 14 [4].

## 3. Raman Spectroscopy

**Figure 1:**
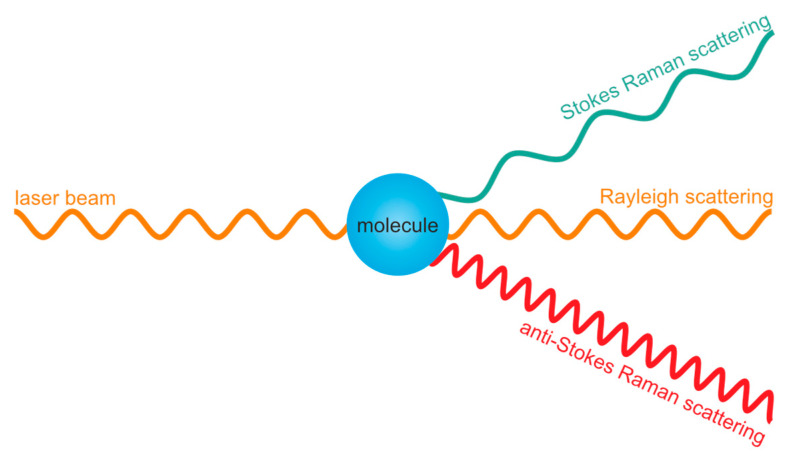
The principle of operation of Raman spectroscopy.

Raman spectroscopy utilizes monochromatic light, usually in the ultraviolet (UV), visible (VIS), and near-infrared (NIR) ranges (200–800 nm wavelength). The source of light is a laser aimed at a sample. The light excites molecules in the sample and undergoes scattering. The vast majority of the scattered photons have the same energy as the source photons (elastic Rayleigh scattering) [40]. Few photons undergo inelastic scattering (with a probability of 1 in 10^8^), known as the Raman phenomenon [41]. The photons are scattered at a shorter frequency (anti-Stokes Raman scattering) or longer frequency (Stokes Raman scattering) than that of the source photons (Figure 1). This energy shift provides information on vibrational states of chemical compounds and their functional groups. The Raman spectra of samples depend on their chemical composition [40,41,42].

Therefore, every molecule and sample has a unique vibrational “fingerprint” [43]. The spectroscopic wavenumber region of cells and tissues ranges from about 500 cm^−1^ to about 1800 cm^−1^. The particular spectral fingerprint of the sample is suitable for characterizing microheterogeneous environments. Collecting numerous spectra from a single sample makes it possible to detect biochemical components and their spatial distribution within the sample. Many conducted studies report that Raman spectroscopy can differentiate between samples sourced from healthy and ill patients and is a sensitive, specific, and overall reliable technique [44].

## 4. The Use of Raman Spectroscopy to Evaluate Various Diseases

### 4.1. Osteoporosis

At present, osteoporosis is recognized as a global public health problem [45]. Osteoporosis is an illness characterized by reduced bone strength and increased fracture risk [46]. An often used indicator of bone strength is bone mineral density (BMD) [47]. Examples of medical conditions leading to a lower BMD are endocrine disorders, chronic kidney diseases, neuromuscular diseases, gastrointestinal diseases, nutritional conditions, menopause, cancer, and HIV. Medications that may cause osteoporosis include glucocorticoids, post-transplant medications, and HIV treatments. Genetic factors also contribute to the risk of osteoporosis occurrence [45]. The standard for diagnosing osteoporosis is taking a measure of the amount of bone mineral via dual-energy X-ray absorptiometry (DXA) [48]. Although DXA is the primary diagnostic tool for osteoporosis, it uses ionizing radiation. Therefore, it is desirable to search for a method that will not expose patients to radiation and will be suitable for the entire population, including children [49,50]. The analysis of keratin molecules in human nails by Raman spectroscopy is a newly proposed tool for the non-invasive evaluation of bone quality and fracture risk [51,52]. Such studies were conducted on adult women’s fingernails [49,51,52,53]; however, considering that osteoporosis also affects children, this new technique could be used in the diagnosis of younger subjects. Such a possibility should be studied, as no publications were found about the application of Raman spectroscopy in examining fingernails of children with suspected bone mineralization disorders. It could lower radiation exposure in children suspected of suffering from osteoporosis.

**Figure 2:**
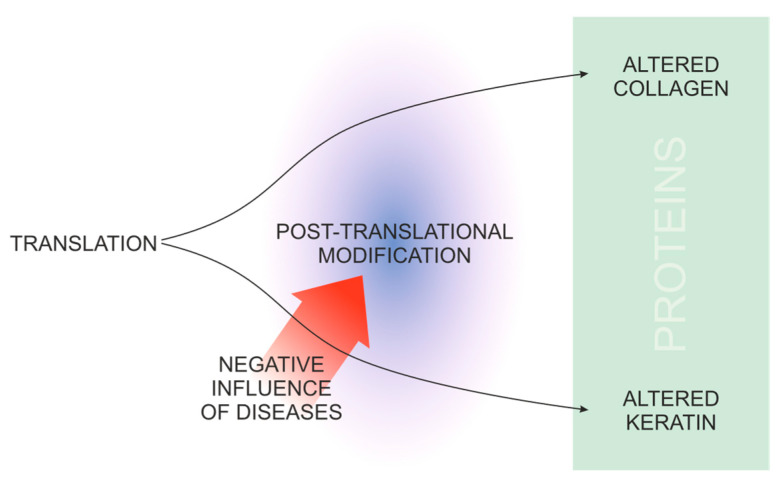
The concept behind the use of Raman spectroscopy in the diagnosis of bone structure disorders.

The first pilot study that looked into the correlation between the keratin structure in fingernails and bone health [54] was based on the fact that both bone collagen and nail keratin are proteins that require sulfation and formation of disulfides. Therefore, any disorder of the mentioned process can lead to the incorrect synthesis of collagen and keratin, resulting in a possible and measurable relationship between nails and bone (Figure 2).

The pilot study was followed by another preliminary study [55]. Participants were divided into two groups by DXA; nine of them were diagnosed as osteoporotic, and thirteen were diagnosed as non-osteoporotic. Obtained Raman spectra for osteoporotic versus non-osteoporotic nails showed that the disulfide bond (S-S) peaks at 510 cm^−1^ were sharper for healthy nails than for osteoporotic nails. Therefore, the disulfide bond content of the nails of healthy patients was higher than that of osteoporotic patients. Even though there were measurable differences in the spectroscopy results of osteoporotic versus non-osteoporotic patients, the small scale of this study made this method inconclusive, and further experiments were required.

In the study of Towler MR et al. published in the same year, 169 volunteers participated [52]. The study aimed to determine whether Raman spectroscopy of nails could be a potential tool to evaluate bone health. A total of 21 patients were assigned to a post-menopausal group and 13 were assigned to a pre-menopausal group. Furthermore, 39 of the 169 subjects reported a history of bone fracture. The S-S peak’s median height was significantly higher (*p* = 0.025) in the non-fracture group when compared to the fracture group, regardless of their menopausal status. When the results were adjusted for age and menopausal status, the S-S peak’s median height was significantly greater (*p* = 0.04) in the non-fracture group when compared to the fracture group. Regression analysis indicated no strong association between the height of the S-S peak and BMD for the lumbar spine of all subjects. Disulfide content in the nails could complement other measures to predict the risk of fracture in patients. Those suggestions were based on the results of the logistic regression analysis. The odd ratio (OR) of the height of the S-S peak was 0.188 95% CI (0.115, 0.307) (*p* < 0.0005) and when BMD was used as a predictor of fracture, the OR was 0.282 95% CI. Those results indicated that both measurements are comparable predictors for fracture risk [52].

Another study enrolled 159 women [49], of whom 81 were pre-menopausal (16 of them had a history of bone fracture) and 78 were post-menopausal (18 of them had a history of bone fracture). It was determined that menopausal status did not significantly influence the scores of the Bone Quality Test (BQT). The BQT is a test that allows identification of sulfur in fingernails, providing information on bone quality [49]. According to previous studies, significantly lower fingernail disulfide content was observed in subjects with a record of bone fracture. Measures of mean lumbar BMD, mean femoral BMD, mean BQT, median osteocalcin, and median CTx were used to differentiate between control and fracture cases. The BQT measurement was the most accurate (*p* = 0.003) discriminator, and the mean lumbar BMD was the second most precise indicator (*p* = 0.009). Results show that BQT performed comparably with DXA, the gold standard for the diagnosis of osteoporosis [49]. Contrary to the previous studies, Mussatto J.C. et al. [50] concluded that there was no relationship between the nail keratin Raman spectrum and osteoporosis evaluated by BMD. In 2014, a study on 213 women (mean age 61.5 ± 9.7 years) was performed. The authors used the peak intensity of the S-S bond (510 cm^−1^) to correlate Raman signatures and BMD of hips and lumbar spine. However, it was pointed out that Raman spectroscopy may be used as an additional tool to diagnose diseases in vivo.

In a study published in 2016 [51] 633 women were enrolled to test the relationship between their current bone health and nail keratin, and to evaluate the sensitivity and specificity of Raman spectroscopy for assessing fracture risk (fracture risk assessment nail correlation (FRAN)). Each patient had to undergo BMD measurements by DXA and provided their history of fractures. The results demonstrated that Raman spectroscopy of fingernails can differentiate between post-menopausal women who have suffered fragility fractures and those who have not. Combining data from Raman spectroscopy with clinical data provides better insight into bone health. The authors conclude that the information provided by Raman spectroscopy of fingernails is distinct from currently available fracture risk predictors. Therefore, combining all these approaches could deliver a new algorithm that outperforms the information obtained from DXA and clinical data alone.

J. R. Beattie et al. published another study in 2017 [56] based on the same group of 633 women as in the previous publication. Its aim was to assess the connection between nail keratin and the bone health of the subjects. In agreement with the prior findings, there was a change in the S-S region of the Raman spectrum. The data suggest an increased amount of broken disulfide bonds in the fracture group, when compared to the non-fracture group. Furthermore, in the fracture cases, there is a noticeable increase in aromatic, aliphatic, acidic, and amide amino acids. No differences were observed between the fracture and non-fracture groups in the case of hydroxyl and basic amino acids. The signals of the non-fracture group results from cysteine, glycine, and serine were more intense. Information about the secondary structure of proteins obtained through Raman spectroscopy shows that the non-fracture controls exhibit a more significant contribution from α-helices and β-sheets. On the contrary, the fracture cases have stronger contributions from random coil of protein. In the osteoporotic vs. healthy comparison, the control group exhibits the presence of α-helices, while the osteoporotic group has a greater share of β-sheets. An opposite pattern was observed in the fracture vs. non-fracture group. A broad band in the disulfide region is present in osteoporosis cases. The authors explained that such broad bands are due to the physical structure of protein becoming more disordered, which indicates increasing variety in local conformations. Therefore, osteoporosis is not associated with the loss of disulfide bonds. The authors proposed that Raman spectroscopy of fingernails represents the status of bone protein structure. They concluded that this technique could offer insight into patients’ bone health and that it is a potential tool to distinguish patients at risk of fracture for reasons other than low BMD.

In another study [53], the same authors used case and control subjects from a 1976 Nurses’ Health Study. They obtained toenail clippings between 1982 and 1984 from 62,865 women. The authors selected 279 post-menopausal subjects at the time of nail collection, aged 50–63, who had hip fractures between 3 and 20 years after the collection of fingernails. Then, a control group from the remaining subjects, who did not report hip fractures, was selected. One control subject per case, with the same month and birth year, was chosen. Overall, the study consisted of 279 case–control pairs of subjects. Raman spectra of fingernails provided information that corresponded with the results of the previous studies. The spectra of the S-S bond for the non-fracture group centered at 510 cm^–1^, and for the fracture group, the S-S presented a weaker and broader peak centered at 525 cm^–1^. The relationship between the Raman scores and the risk of hip fracture in unconditional logistic regression models controlled for age was examined. The time between the sample collection and fracture occurrence was also taken into consideration. Hip fracture prediction up to 20 years based on the Raman score achieved an odd ratio (OR) of 2.2 (95% CI). The predictability of fracture based on clinical risk factors (CRF) reached an OR similar to the Raman score; the highest predictability was achieved for CRF and the Raman score altogether, with an OR of 3.8 (95% CI). The authors stated that Raman spectroscopy can be a promising tool to identify post-menopausal women younger than 65 years with an increased risk of hip fracture up to 13 years. The preliminary results of this study are promising when compared to existing predictive technologies (DXA, CRF, quantitative ultrasound).

The available data on use of Raman spectroscopy in bone mineralization disorder diagnosis are summarized in the Table 1. To our knowledge, in the pediatric population, Raman spectroscopic analysis has been used only to establish reference data of trabecular bone quality for children and young adults. Significant dependencies of the measured parameters on tissue age were found, while sex and subject’s age were not confounders at any given tissue age [57].

### 4.2. Onychomycosis

Onychomycosis is the most common infection of the nail [59,60]. In children, dermatophyte onychomycosis occurs rarely, more often after the age of six, and is even more frequent among adolescents [61,62]. The nail plate in children shows faster linear growth than in adults (and, thus, faster fungus elimination). It is also rare for a child to have impaired circulation, as often seen in adults, which favors fungal infections of the nails.

Moreover, children attend public places less often, which reduces the risk of fungal infection [63]. However, the following groups of pediatric patients are more likely to have fungal nail infections: children with Down syndrome, immune deficiencies, and children suffering from immunosuppression [64,65]. In recent years, an increase in the incidence of onychomycoses has been reported in the pediatric population. Importantly, patients with onychomycosis should be attentively examined for concomitant mycoses in other areas [66].

Wei Wen et al. [2] used Raman spectroscopy to analyze keratin structural changes in human adult fingernail samples affected by onychomycosis. They established that nails from patients with onychomycosis had a significant reduction in the content of the sulfur-containing amino acids in comparison to healthy nails. Moreover, the spectral analysis demonstrated a significant increase in the relative contributions of energetically less stable disulfide linkages (gauche–gauche–trans forms). On the other hand, the content of α-helix and the random coil remarkably decreased in nail clippings affected with onychomycosis when compared to normal nails. These findings reflected the molecular structure changes in nail keratins seen in fungal erosion. They could be useful as a diagnostic tool (to differentiate the normal and infected nails by a non-invasive method) and for follow-up during the course of onychomycosis treatment [2].

Early and accurate diagnosis of onychomycosis with identification of the causative species is the key factor for optimal therapy. The current gold standard for the diagnosis of onychomycosis, and for the differentiation of the infectious agents, is a direct microscopic evaluation of nail clippings and cultivation of affected nail samples in specialized media [67]. Direct microscopy is inexpensive and easy, but its sensitivity depends highly on the operator [68]. The growth of dermatophytes in media is slow and usually requires 3–4 weeks of incubation, so the search for a rapid, non-destructive, and non-invasive method of their identification (especially in children) is understandable. It is also essential to differentiate between dermatophytic and non-dermatophytic agents because it determines treatment options, efficacy, and prognosis [69]. Smijs et al. [70] used Raman spectroscopy in an ex vivo onychomycosis model (infection of nail clippings) to differentiate between *Trichophyton* dermatophytic fungi *(T. rubrum*, *T. mentagrophytes*, and *T. tonsurans*) and the non-dermatophytic species (*S. brevicaulis* and *C. albicans*). Raman spectra were analyzed by sorting the correlation matrix. They were presented as a dendrogram which illustrated cluster arrangements with the dermatophytic nail infections in one cluster and the non-dermatophytic/yeast nail infections in a separate one. Spectral dissimilarities between tested dermatophytes were also found, with *T. rubrum* being the most distinctive [70]. A few years later, Kourkoumelis et al. [71] confirmed that Raman spectroscopy could be a method for the differentiation of healthy versus diseased nails, including the specific distinction between onychomycosis caused by *T. rubrum* and *Candida* species. Petrokilidou et al. [72] demonstrated additionally that treatment of nail clippings with ethyl alcohol improves the efficacy of Raman spectroscopy in the recognition of onychomycosis, especially caused by *T. rubrum*. The authors proposed this technique as a simple and rapid method for detecting onychomycosis. In this method, an optical fiber can be directly positioned on the infected nail; therefore, there is no need to perform a nail biopsy. It can be easily integrated in the clinical setting to provide real-time and on-site evaluation of diseased nails [66,70]. Therefore, this non-invasive technique is a promising method, especially in pediatric patients with a higher risk of onychomycosis.

Raman spectroscopy was also used to explore the changes in the chemical structure of nail keratin before and after onychomycosis treatment with a 1064 nm Nd:YAG laser [73]. After laser treatment, the disulfide band (490–590 cm^−1^) was prominently decreased and the amide I band (1500–1700 cm^−1^) was altered. In the treated nails, the β-sheet structure (1672 cm^−1^) was more dominant than the α-helical one (1652 cm^−1^).

The available data on the use of Raman spectroscopy in onychomycosis treatment are summarized in the Table 2.

### 4.3. Psoriasis

Psoriasis is an inflammatory disease associated with immune system dysfunction that results from a polygenic predisposition combined with environmental triggers, e.g., trauma, infections, medications, and psychological stress [76]. It affects about 2–3% of the world’s population, with equal sex incidence [77]. Approximately 10–78% of patients with psoriasis also suffer from nail psoriasis [78]. Fingernails are more commonly affected because they grow faster than toenails [79]. Hyperproliferation and differentiation of keratinocytes due to abnormal immune response affect the nail matrix, nail bed, and hyponychium. Small parakeratotic foci in the proximal matrix cause nail pitting, and involvement of the intermediate or ventral matrix leads to leukonychia. The so-called “oil drop” or “salmon patch” phenomenon is also a common psoriatic manifestation, reflecting exocytosis of leukocytes beneath the nail plate. Psoriasis increases capillary fragility, which leads to splinter hemorrhages [80,81,82].

If nail psoriasis is accompanied by skin psoriasis, diagnosis is relatively simple. However, up to 10% of nail psoriasis cases occur independently and can be difficult to diagnose [78]. The prevalence of nail psoriasis in Caucasian pediatric patients was evaluated to be 10.2% [83]. Various diagnostic methods are used in such cases, including nail biopsy, dermoscopy, capillaroscopy, optical coherence tomography, and confocal laser scanning microscopy [76]. Chiriac et al. used Raman spectroscopy to find microstructural alterations in psoriatic nails [84]. The Raman spectra of psoriatic nails provided information on the functional groups of compounds building them and allowed for comparison of the chemical composition between healthy and psoriatic nails. The authors concluded that psoriasis is much more aggressive than onychomycosis, affecting the S-S bond through sulfonic group formation. Moreover, the results suggest that the α-helical structure of keratin is partially destroyed by psoriasis [84]. Cutrin Gomez et al. [75] also analyzed psoriatic nails samples by the Raman method. They noticed a significant reduction in the S-S to C-C signals ratio for psoriatic nails, suggesting the presence of broken disulfide bonds.

The difficulty of diagnosing isolated childhood nail psoriasis is even greater, due to the similarity of its most typical symptoms (nail pitting, onycholysis associated with subungual hyperkeratosis, and pachyonychia and paronychia) to those of other diseases prevalent in children (onychomycosis, trachyonychia, eczema of the nails, traumatic nail dystrophies, alopecia areata) [83]. Each of these diseases requires a different therapeutic approach. Nail psoriasis can have a serious psychological impact on affected individuals, and it can be a major functional problem, especially for children and youth. They often withdraw from public life due to the cosmetic defect, pain, and discomfort [85,86,87]. The development of reliable and fast differentiating methods in nail dermatology, such as Raman spectroscopy, could expedite the diagnostic process and be better tolerated by pediatric patients thanks to their non-invasive character. This also could result in the rapid implementation of appropriate treatment.

The available data on the use of Raman spectroscopy in psoriasis are summarized in the Table 3.

### 4.4. Malignant Melanoma

Malignant melanoma (MM) is the most aggressive skin cancer and is invariably fatal if left untreated. Difficulties in diagnosis of cutaneous MM arise because benign lesions may resemble melanoma [88,89]. It is crucial to diagnose it accurately as early as possible; however, on the other hand it is important to avoid numerous unnecessary biopsies of benign lesions. Clinical diagnostic accuracy of early melanoma is still low; therefore, improved non-invasive tools are required for detection of MM. Despite attempts to use methods, such as total body photography, dermatoscopy, high-frequency skin ultrasonography, fluorescence spectroscopy, positron emission tomography, no credible technique for early diagnosis of MM has been established [90]. Raman spectroscopy has also been tested in this area. It was proven that this technique can be applied in cell cultures to distinguish between normal melanocytes and melanoma cells, to observe the course of cell death in MM cells, and to examine their susceptibility to anticancer drugs [91]. Ex vivo studies have shown a possibility to differentiate cutaneous melanoma from pigmented nevus [92], and melanoma from its metastasis and normal skin [93]. Analysis of skin MM sample spectra suggested an alteration in protein structure. A decrease in the amide I and amide III bands can be seen. Lipid band intensity was increased, on the other hand [90]. Santos et al. has shown a presence of distinctive differences between MM and benign melanocytic lesions spectra in regions attributed to lipids, too [94]. In vivo studies on use of Raman spectroscopy in MM diagnosis were also conducted. Harvey et al. were able to distinguish malignant MM skin lesions from benign ones (pigmented skin lesions and seborrheic keratoses). The diagnostic accuracy of Raman spectroscopy was deemed to be comparable to clinical examination and other optical methods [95]. Philipsen et al. noticed that Raman-based diagnostic methods can be applied to patients regardless of the level of their skin pigmentation [96]. The results of in vivo studies agreed with the results of previously cited ex vivo studies (alternations of lipid and protein structure). Not only was the method useful to differentiate between malignant and benign melanocytic lesions, but it also performed well in differentiation between malignant melanoma and malignant non-melanoma skin cancers [97]. In 2018, a meta-analysis of existing data was conducted. It proved the effectiveness and accuracy of Raman spectroscopy in this field. The method was proposed as an intraoperative tool for precise determination of tumor margin. Zhang et al. claimed that Raman spectroscopy is a promising screening method in clinical practice which may reduce the number of unnecessary biopsies. What is more, it is fast and inexpensive [98,99].

Nail unit melanoma (NUM) is a specific form of MM. It represents 1–2.5% of all diagnosed melanomas in white, 15–35% in dark-skinned, and 50% to 58% in Asian populations. NUM localizes either under (subungual) or around (periungual) the nail and is categorized as a variant of acral lentiginous melanoma (ALM) [100]. Unfortunately, it is seldom diagnosed at early stages. Typical symptoms of NUM (such as longitudinal melanonychia) are not characteristic to this disease and require broad differential diagnostics. Some of the early symptoms (irregular pattern of the longitudinal microlines and periungual pigmentation, also known as the micro-Hutchinson sign) are only dermoscopically visible [101,102]. Due to the poor general knowledge of the issue, early symptoms may go unnoticed both by clinicians and patients. Advanced stages of NUM are characterized by more prominent symptoms. In the light of this, patients are at high risk of misdiagnosis, and the proper diagnosis is made too late [103].

NUM also occurs in children; however, it is rare in the pediatric population [104]. Diagnosis of children is especially difficult due to sparse evidence in publications and ongoing discussions, which results in lack of clear differentiating and diagnostic criteria. Benign lesions in children exhibit some similarities with malignant lesions in adults, which makes deciding even more challenging [102]. Due to the issues described above, any additional tool would improve the chances of proper diagnosis of NUM. Raman spectroscopy is a promising, non-invasive, rapid method of skin melanoma detection. Therefore, it is worth considering the application of this method to analyze suspicious nail lesions. Up to this point, we found no papers on use of Raman spectroscopy in NUM research. It seems to be a challenge that should be tackled in the near future to better the chances for survival of malignant melanoma patients, with particular reference to nail unit melanoma.

### 4.5. Diabetes Mellitus

Globally, diabetes affected a population of 424.9 million people in 2017, and its prevalence is still increasing, with an estimated 628.6 million people likely to be affected by 2045. Diabetes usually affects people between 40 and 59 years of age [105]. In Poland, the estimated prevalence of diabetes is 6.97% [106]. In 2010, in Germany, there were approximately 32,000 patients under the age of 20 years old with type 1 diabetes. The incidence is rising by 3–4% each year. Yearly, there are 22.9 new cases per 100,000 patients up to the age of 15 years old [107]. A multicenter cohort study from 2017 [108] showed that the incidence of type 1 diabetes during 5-year observation period increased 1.5-fold in children living in eastern and central Poland, with the most significant increase occurring in children aged 10 to 14 years, and with a significantly higher increase in children living in cities compared to children living in rural areas.

Diabetes diagnostics is based on strictly defined protocols for blood glucose testing in venous and capillary blood. Currently, glycated hemoglobin (HbA1c) is a very valuable indicator of diabetes, not only for diagnosis but also for controlling the course of the disease. However, HbA1c level may be influenced by various hemoglobinopathies (e.g., thalassemia), factors that impact erythrocyte survival, hyperbilirubinemia, uremia, and iron deficiency [109]. The development of new innovative diagnostic methods is inevitable due to economic reasons and due to the need for non-invasive solutions [110].

The growth of the nail plate is slow enough for it to be an essential material to evaluate the long-term effects of hyperglycemia on tissue quality [111]. Since nail protein glycation was proposed as a marker in diabetes more than 30 years ago [112], it seems reasonable that physical methods, like Raman spectroscopy, could be valuable tools. It has been proved that Raman spectroscopy can distinguish between spectra generated by glycated and non-glycated forms of hemoglobin [113].

Farhan KM et al. [114], using Fourier transform infrared spectroscopy (FTIR), showed that the diabetic patients’ nail protein differed from non-diabetic ones. In the study, the diabetic nail samples showed the amide I band at 1645–1659 cm^−1^, where 1650 cm^−1^ corresponds to amide I of α-helical structures. Amide II bands are observed around 1540 cm^−1^. Amide III bands are observed around 1259 cm^−1^. In the case of all non-diabetic fingernail samples the amide I band is observed below 1640 cm^−1^. The amide II bands are absent in non-diabetic specimens.

The homeostasis of trace elements can be affected by diabetes mellitus [115], as chromium, magnesium, vanadium, zinc, manganese, and selenium play roles in insulin action [116]. Deficiencies of certain minerals, including Mg, Zn, and Cr, predispose individuals to glucose intolerance and promote the development of diabetic complications consisting of retinopathy, thrombosis, hypertension [117,118,119], impaired repair of tissues and wound healing [120,121], and diabetic angiopathy [118,122]. 

Esfahani et al. [123] assessed trace element levels (zinc, copper, magnesium, selenium, manganese, and chromium) in biological samples (including toenails) of 150 diabetes type II patients and 151 non-diabetic subjects by inductively coupled plasma atomic emission spectroscopy (ICP-AES). Authors discovered that nail and hair zinc and chromium concentrations were significantly lower in people with diabetes than non-diabetics. There were significant differences between the two groups in terms of nail selenium and hair manganese concentrations. The results confirmed that nail samples are excellent biological samples for trace element spectral evaluation, specifically in the case of chromium, selenium, and manganese, due to the excessive accumulation of these elements in nails and hair.

Monteyne et al. [124] used near-infrared (NIR) spectroscopy for the assessment of diabetes mellitus. Their results suggested that analysis of glycated nail proteins by NIR spectroscopy could serve as a diagnostic marker of diabetes mellitus, not only as an alternative and affordable tool but also in situations where blood sample analysis is impossible. This monitoring technique is painless and non-invasive, which could benefit pediatric patients.

## 5. Conclusions

Raman spectroscopy is a promising, non-invasive, and safe method for evaluating bone quality and fracture risk in adult populations. In nail dermatology, Raman spectroscopy can detect onychomycosis, differentiate infectious agents (dermatophytic fungi and non-dermatophytic species), and find microstructural alterations in psoriatic nails. Raman spectroscopy is also a reliable tool for skin melanoma detection because it allows differentiation between malignant and benign melanocytic lesions and between malignant melanoma and malignant non-melanoma skin cancers. 

Unfortunately, there are no papers on use of this method in nail unit melanoma detection. Nail unit melanoma diagnosis is exceptionally challenging, and any new method would aid clinicians in their efforts. Data about the application of Raman spectroscopy in the examination of the nails of children with suspected bone mineralization disorders, onychomycosis, psoriasis, melanoma, and diabetes mellitus are also limited. Therefore, more studies are required on children to ensure a precise and less stressful diagnostic process.

## Figures and Tables

**Table 1:** A summary of available original articles addressing the use of Raman spectroscopy in the diagnosis of bone mineralization disorders.

Study	Study Population	Results and Conclusions
[54,55]	Control group n = 13 Osteoporosis group n = 9	Lower nail disulfide content in the osteoporosis group than in the control group. Relationship between the nail and bone content of disulfide bonds was observed.
[52]	Pre-menopausal group n = 84 (including bone fracture group n = 18) Post-menopausal group n = 85 (including bone fracture group n = 21)	Difference in the disulfide content of nails sourced from pre- and post-menopausal women. Lower disulfide content in nails sourced from women with a history of fracture independent of menopausal status. A promising method for examination of fracture risk.
[58]	Patients undergoing hormonal therapy for early breast cancer n = 13 (including tamoxifen treatment n = 4; letrozole treatment n = 9; bone fracture n = 8)	Reduction in BQT scores after a six-month period regardless of the treatment method.
[49]	Pre-menopausal group n = 81 (including bone fracture group n = 16) Post-menopausal group n = 78 (including bone fracture group n = 18)	Lower nail disulfide content in subjects with a history of fracture. BQT discriminated most accurately between the control and fracture cases. A promising method for the examination of fracture risk.
[50]	Normal BMD n = 208 (including measurements taken at hip n = 109 and lumbar n = 99) Osteopenia n = 178 (including measurements taken at hip n = 93 and lumbar n = 85) Osteoporosis n = 40 (including measurements taken at hip n = 11 and lumbar n = 29)	No correlation was found between the peak intensities of the S-S bonds and the BMD values of hip and lumbar. No relationship between the nail keratin Raman spectrum and the osteoporotic disease assessed by BMD.
[51]	Post-menopausal women n = 633 (including subjects with and without bone fracture history)	Differences in nail protein structure between fracture and non-fracture groups. Raman spectroscopy could be included in fracture risk assessment protocols.
[56]	Post-menopausal women n = 633 (including bone fracture and non-bone fracture groups and osteoporosis and no osteoporosis groups)	Altered nail keratin structure and different amino acid composition of the nail in the fracture group. Lack of statistical significance in differentiation of osteoporosis cases.
[53]	Post-menopausal group without history of fracture n = 81 Post-menopausal group with history of fracture n = 82	Raman spectra suggest a decrease in disulfide bonds and an increase in -SH groups of proteins. A promising method for the examination of fracture risk.

**Table 2:** A summary of the available original articles addressing the use of Raman spectroscopy in the diagnosis of onychomycosis.

Study	Study Population	Results and Conclusions
[2]	Control group n = 8 Onychomycosis group n = 5	Reduction in sulfur-containing amino acids content in nails obtained from patients with onychomycosis in comparison the control group. A trend to the energetically less-favored g–g–t form in the disulfide bonds in the nails of patients with onychomycosis. Raman spectroscopy may become a tool used in diagnosis of nail fungal infection.
[70]	No data on the number of subjects whose nails were analyzed. A total of 18 nail samples were infected ex vivo with *T. rubrum*, *T. mentagrophytes*, *T. tonsurans*, *S. brevicaulis*, or *C. albicans*.	Raman spectroscopy can be used for differentiation between infections caused by dermatophytes (*T. rubrum*, *T. mentagrophytes*, *T. tonsurans*) and non-dermatophytes (*S. brevicaulis*, *C. albicans*). A promising method for examination of pediatric patients.
[71]	Healthy control n = 26 *T. rubrum* infection group n = 12 *Candida* infection group n = 14	Differentiation between healthy nails, nails infected with *T. rubrum*, and nails infected with *Candida* species is possible with use of Raman spectroscopy.
[72]	Participants n = 15 (including some with suspected onychomycosis)	Ethyl alcohol improves the efficacy of *T. rubrum* detection in nails with the use of Raman spectroscopy.
[73]	*T. rubrum* infection group n = 3	Nail keratin structure changes during Nd:YAG laser treatment.
[74]	No data on the number of subjects whose nails were analyzed. Samples were obtained from healthy individuals and patients with onychomycosis. Nail samples infected ex vivo were used too.	No increase of amount of -SH groups observed in the infected nails.
[75]	No data on the number of subjects whose nails were analyzed. Samples were obtained from healthy individuals, patients with onychomycosis, and patients with psoriasis.	Onychomycosis causes a reduction in the number of S-S bonds in the nails to a greater extent than psoriasis.

**Table 3:** A summary of available original articles addressing the use of Raman spectroscopy in the diagnosis of psoriasis.

Study	Study Population	Results and Conclusions
[84]	Control group n = 7 Nail psoriasis n = 18 (including patients n = 16 and patients treated with biologics n = 2)	Psoriasis affects surface morphology of nails. Keratin’s α-helix structure is partially destroyed in psoriasis and S-S bonds are broken. Biological treatment leads to the reformation of S-S bonds.
[75]	No data on the number of subjects whose nails were analyzed. Samples were obtained from healthy individuals, patients with onychomycosis, and patients with psoriasis.	Psoriasis causes a reduction in the number of S-S bonds in the nails, but to a lesser extent than onychomycosis.

## Data Availability

Not applicable.
